# Clinical Characteristics, Treatment Strategy, and Outcomes of Primary Large Cell Neuroendocrine Carcinoma of the Bladder: A Case Report and Systematic Review of the Literature

**DOI:** 10.3389/fonc.2020.01291

**Published:** 2020-07-28

**Authors:** Kun Xia, Wenlong Zhong, Junyu Chen, Yiming Lai, Guohui Huang, Hao Liu, Wen Dong, Wang He, Tianxin Lin, Jian Huang

**Affiliations:** ^1^Department of Urology, Sun Yat-sen Memorial Hospital, Sun Yat-sen University, Guangzhou, China; ^2^Department of Pathology, Sun Yat-sen Memorial Hospital, Sun Yat-sen University, Guangzhou, China

**Keywords:** carcinoma, neuroendocrine, large cell, bladder cancer, systematic review

## Abstract

**Purpose:** The aim of this study was to review the clinicopathologic characteristics, treatments, and outcomes of patients with primary large cell neuroendocrine carcinoma of the bladder (LCNEC).

**Patients and Methods:** We report one patient diagnosed with primary pure LCNEC of the bladder in Sun Yat-sen Memorial Hospital. In addition, we performed a systematic literature review, in April 2020, on case report and case series of LCNEC of the bladder. The clinicopathologic characteristics, treatments and outcomes of this rare disease were analyzed.

**Results:** A total of 39 patients were included in our analysis (1 case from our institution and 38 cases from the literature). Most patients (79.5%) were male. The average age at the surgery for the patients is 61.5 years (range 19–85 years). The most common symptom was hematuria (*n* = 20, 76.9%). Almost all patients (38, 97.4%) underwent surgery, with 26 (66.7%) receiving multimodality therapy. Out of 24 patients with available data, regional or distant recurrences developed in 14 patients (58.3%). The median overall survival of the patients was 11.5 months, with 1- and 3-year survival rates of 54.0 and 21.4%, respectively. In the survival analysis, theT1–2 tumors (*P* = 0.025), no distant metastases at diagnosis (*P* = 0.001), and multimodality therapy (*P* = 0.017) were associated with better overall survival (OS).

**Conclusions:** LCNEC of the bladder is an extremely rare neoplasm. The available data suggest that the disease has an aggressive natural history with poor prognosis. Early pathologic stage and multimodality treatment may be important factors in determining prognosis.

## Introduction

Urinary bladder cancer (BCa) is the 10th most common malignancy cancer worldwide, with an estimated 549,393 new cases and 199,922 deaths in 2018 (1). However, non-urothelial cancers of the urinary bladder are relatively rare, accounting for only 5% of all BCa (2, 3). Moreover, primary bladder neuroendocrine carcinoma is an extremely rare but heterogeneous variation of non-urothelial carcinoma of the urinary bladder, representing <1% of urinary bladder neoplasms. According to the 2016 WHO classification of bladder tumors, bladder neuroendocrine carcinoma includes small cell neuroendocrine carcinoma, large cell neuroendocrine carcinoma (LCNEC), paraganglioma, and well-differentiated neuroendocrine tumor (4). Among the four subtypes, the most common subtype is small cell neuroendocrine carcinoma, while LCNEC is exceedingly rarely (5, 6). Although first recognized by Abenoza et al. more than 30 years (7), there are few available data for LCNEC of the bladder.

Due to the rarity of LCNEC of the bladder, the biological, and clinicopathological characteristics remain largely elusive. Current knowledge of this disease is mainly based on small series and case reports. No consensus has been reached in standard treatment strategy for patients with LCNEC of the bladder.

In the present study, we reported a case of primary pure LCNEC of the bladder at our tertiary center. To our knowledge, the present study reported the first case of primary pure LCNEC of the bladder among Chinese patients. In addition, to achieve better understanding of the disease, we performed a systematic review with an attempt to describe the clinicopathologic characteristics and treatment strategy of LCNEC of the bladder.

## Patients and Methods

### Case Presentation

A 39-year-old man was referred to our hospital with painless gross haematuria for 6 days. No palpable mass was found on physical examination. The patient had no history of cigarette smoking. The pelvis CT revealed a suspicious mass addressing the anterior right wall of bladder (41 mm × 34.8 mm × 31.0 mm) with multiple high-density calcification lesions (Supplementary Figure 1). The patient received a diagnostic trans-urethral resection of the bladder tumor (TURBT) and the mass was diagnosed with primary LCNEC of the bladder.

**Figure 1:**
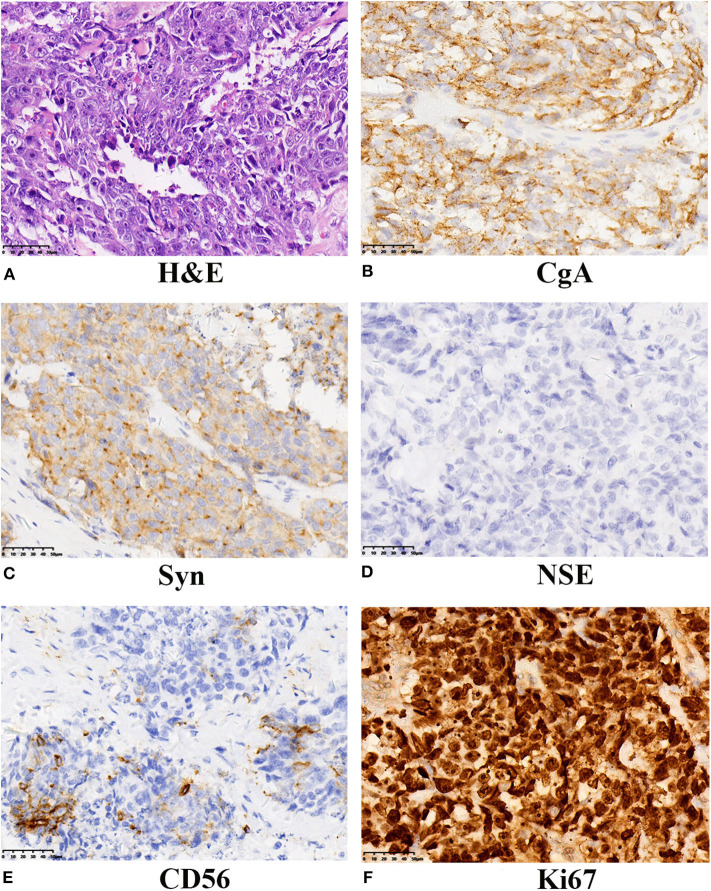
Histochemical and immunohistochemical examinations of the tumor from our case. **(A)** LCNEC of the bladder showed large cells with large nuclear size, prominent nucleoli, and abundant cytoplasm. (H&E staining, ×400). The neoplasm displayed positive expression of CgA **(B)**, strong and diffused expression of Syn **(C)**, absent NSE expression **(D)**, positive for CD56 **(E)**, and the proliferative index Ki67 was 90% **(F)**. A final diagnosis of LCNEC was performed. LCNEC, large cell neuroendocrine carcinoma; H&E, hematoxylin and eosin; CgA, Chromogranin A; Syn, synaptophysin; NSE, neuron specific enolase.

Subsequently, the patient underwent radical cystectomy and lymph node dissection 1 week later. The final pathological report of radical cystectomy confirmed the diagnosis of primary pure LCNEC without lymph node involvement. The final pathological stage was T2bN0M0. As seen in Figure 1, immunohistochemical analysis showed the neoplastic cells were positive for neuroendocrine markers like: chromogranin A (CgA), synaptophysin (Syn), and CD56. The expression of cellular proliferation marker Ki-67 was up to 90%.

After surgery, the patient received a subsequent 5-cycle adjuvant chemotherapy with etoposide and cisplatin. The patient is alive without any evidence of recurrence during 59-month follow-up.

### Search Strategy

A literature search was performed using the PubMed and Embase database to identify all full text available English articles on LCNEC of the bladder, before April 2020. The search terms “Bladder” and “LCC” or “Large Cell Carcinoma” or “Neuroendocrine” were used. The reference lists of the relevant articles were also searched for additional cases. Two independent reviewers identified the studies and disparities were resolved with a third reviewer. Data involving name of first author, year of publication, patient demographics, clinical characteristics, radiographic and pathological results, and therapeutic management were extracted.

### Statistical Analysis

Descriptive data were presented as frequency and percentages. The overall survival (OS) was analyzed using the Kaplan–Meier method, and the statistical significance was determined using the log-rank test. Univariate analysis was calculated using Cox proportional hazards regression. Statistical analysis was performed using the SPSS software version 25.0 (IBM Corporation, Armonk, NY, USA) and GraphPad Prism software (Graph-Pad Software, Inc., La Jolla, CA, USA). A 2-sided *P* < 0.05 was taken to indicate statistical significance.

## Results

In total, 25 publications (6–29) involving 38 cases were identified. The unreported case in our study was also included. Overall, a total of 39 patients with LCNEC of the bladder were enrolled.

### Clinical and Pathological Characteristics

Data including demographics, pathological characteristics, and treatment are summarized in Table 1. The mean age of patients was 61.5 years (range 19–85 years) with a male-female ratio nearly 4:1 (31 vs. 8). Most patients were Caucasian (*n* = 32, 82.1%). The most commonly reported symptoms were hematuria (*n* = 20, 76.9%). Eight out of 15 patients (53.3%), for which the information was recorded, had a history of smoking.

**Table 1:** The baseline of clinicopathologic characteristics and therapy of patients.

**Characteristics**	***N* (%), median (range)**
**Age (34 available) (years)**	61.5 ± 17.9 (19-85)
≥65	18 (52.9)
<65	16 (47.1)
**Gender (39 available)**
Male	31 (79.5)
**Female**	8 (20.5)
**Race (39 available)**
Caucasian	32 (82.1)
Asian	7 (17.9)
**Histology (39 available)**
Pure	22 (56.4)
Mixed	17 (43.6)
**Tumor size (cm) (16 available)**	4.11 ± 1.76 (1.0–9.1)
**TNM stage (30 available)**
I–II	9 (30.0)
III–IV	21 (70.0)
**Pathologic stage (30 available)**
T1–2	10 (33.3)
T3–4	20 (66.7)
**Pathologic node status (22 available)**
N0	14 (66.7)
N+	7 (33.3)
**Distant metastasis at diagnosis (24 available)**
M0	18 (75.0)
M+	6 (25.0)
**Surgery (38 available)**
RC	27 (71.1)
PC	4 (10.5)
TURBT	7 (18.4)
**Adjuvant chemotherapy (39 available)**
Yes	24 (61.5)
No	15 (38.5)
**Radiotherapy (39 available)**
Yes	8 (20.5)
No	31 (79.5)

*RC, radical cystectomy; PC, partial cystectomy; TURBT, transurethral resection of bladder tumor*.

With regard to the tumor characteristics, the mean tumor size was 4.11 cm (range 1.0–9.1 cm). Only 1 patient was classified as non-muscle invasive bladder cancer. Moreover, 33.3% (7/21) and 25% (6/24) patients were clinically diagnosed with lymph node invasion and metastatic disease at the time of diagnosis, respectively. Out of 30 patients with available data, 10 (33.3%) were classified as T1–2 while 20 (66.7%) were classified as T3–4 tumors. For 17 cases (43.6%), the pathological components were coexisted with urothelial carcinoma (UC; *n* = 10, 25.6%), small cell carcinoma (SCC) (*n* = 4, 10.3%), adenocarcinoma (*n* = 4, 10.3%), squamous cell carcinoma (*n* = 1, 2.6%), carcinosarcoma (*n* = 2, 5.1%), and lymphoma (*n* = 1, 2.6%).

### Treatment and Prognosis

Among the 39 patients, all except one case underwent surgery, with 26 (68.4%) undergoing radical cystectomy (RC), 8 (18.4%) undergoing TURBT and 4 (10.5%) undergoing partial cystectomy. Noteworthy, 26 (66.7%) patients received multimodality therapy. Among the patients treated with multimodality therapy, 61.5% received platinum-based adjuvant chemotherapy. Three patients were given neoadjuvant chemotherapy and 8 patients underwent radiotherapy.

The mean follow-up time was 17.5 months. Out of 27 patients with available data, 14 patients (51.9%) developed disease recurrence. Ten out of 24 patients (41.7%) developed distant metastases after surgery with a mean time of 7.8 months. The most common sites of metastasis were distant lymph nodes (*n* = 5, 50%), lung (*n* = 4, 40%), and liver (*n* = 3, 30%). Once the metastases occur, the average survival time was <3 months.

**Figure 2:**
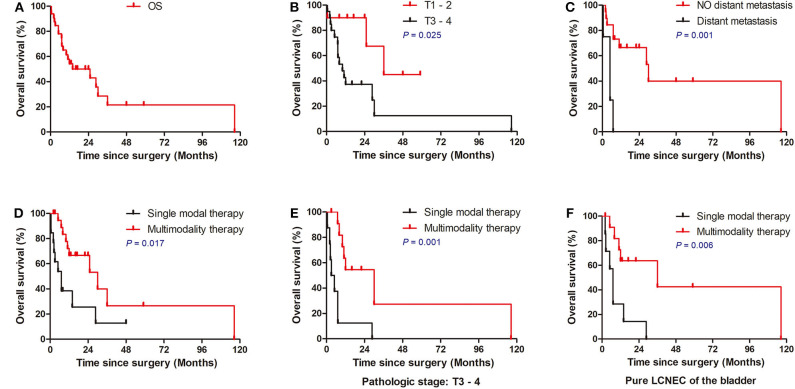
Overall Kaplan–Meier survival curves for patients with LCNEC. **(A)** Survival of all patients. **(B)** Survival of patients stratified according to TNM stage. **(C)** Survival of patients stratified according to T stage. **(D)** Survival of patients stratified according to M stage. **(E)** Multimodality therapy vs. single therapy according to pure LCNEC of the bladder. **(F)** Multimodality therapy vs. Single therapy according to pathologic stage T3–4. LCNEC, large cell neuroendocrine carcinoma; ACT, adjuvant chemotherapy.

The median overall survival was 11.5 months, with 1- and 3-year survival rates of 54.0 and 21.4%, respectively (Figure 2A). In the cox univariate analysis (Table 2), advanced pathologic T stage (T3–4) (*HR*, 3.886; 95% CI 1.085–13.913; *P* = 0.037), distant metastasis at diagnosis (*HR*, 8.392; 95% CI 2.240–31.434; *P* = 0.002), without adjuvant chemotherapy (*HR*, 2.52; 95% CI 1.002–6.333; *P* = 0.049), and single modal therapy (*HR*, 2.884; 95% CI 1.156–7.193; *P* = 0.023) were the risk factors of poor OS. Of note, no statistical difference was observed in OS between the RC group and bladder-sparing group (*HR*, 1.781; 95% CI 0.685–4.634; *P* = 0.237). All clinical and pathologic variables were well-balanced between the 2 groups (Supplementary Table 1). Kaplan–Meier survival analysis confirmed that T1–2 tumors (median OS: 23.9 vs. 16.0 months, *P* = 0.025), no metastasis at diagnosis (median OS: 22.9 vs. 5.2 months, *P* = 0.001) and multimodality therapy (median OS: 22.2 vs. 10.4 months, *P* = 0.017) were associated with better OS (Figures 2B–D). There was no difference in OS between the pure and mixed LCNEC (*HR*, 1.110; 95% CI 0.436–2.831; *P* = 0.826). All clinical and pathological variables were well balanced between the pure and mixed LCNEC groups (Supplementary Table 2). Kaplan–Meier survival analysis confirmed the similar OS between the pure LCNEC and mixed LCNEC groups (Supplementary Figure 2).

**Table 2:** Univariate analysis of prognostic factors for OS of the patients.

**Characteristics**	***N* (*n* of death)**	**Univariate analysis**		
		**HR**	**95% CI**	***P*-value**
**Age (years)**		0.531	0.208–1.356	0.186
≥65	18 (12)			
<65	15 (8)			
**Gender**		1.053	0.375–2.961	0.922
Male	26 (15)			
Female	7 (5)			
**Ethnic**		3.492	0.799–15.254	0.096
Caucasian	26 (18)			
Asian	7 (2)			
**Histology**		1.11	0.436–2.831	0.826
Pure	19 1(3)			
Mixed	14 (7)			
**Tumor size (cm)**		0.507	0.091–2.828	0.439
≤ 4	7 (2)			
>4	8 (3)			
**Smoking**		1.205	0.229–6.332	0.826
Yes	6 (4)			
No	8 (3)			
**Pathologic stage**		3.886	1.085–13.913	0.037^*^
T1–2	10 (3)			
T3–4	20 (15)			
**Pathologic node status**		3.037	0.919–10.041	0.069
N0	14 (6)			
N+	7 (6)			
**Distant metastasis at diagnosis**		8.392	2.240–31.434	0.002^*^
M0	18 (7)			
M+	6 (6)			
**Surgery**		1.781	0.685–4.634	0.237
RC	21 (12)			
Bladder-sparing surgery	11 (7)			
**Adjuvant chemotherapy**		2.52	1.002–6.333	0.049^*^
Yes	18 (9)			
No	15 (11)			
**Multimodality therapy**		2.884	1.156–7.193	0.023^*^
Yes	20(10)			
No	13 (10)			

* *P-value, 0.05. RC, radical cystectomy; OS, overall survival. Bold values are statistically significant (p < 0.05)*.

In the exploratory subgroup analysis, we investigated the role of treatment strategy in subgroups stratified according to histology and pathologic T stage. Compared to patients receiving single modal therapy, patients received multimodality therapy had significantly better OS in with the subgroup of pure LCNEC of the bladder (*P* = 0.006; Figure 2E) and advanced pathologic T stage (*P* = 0.001; Figure 2F). However, no significant difference was observed in patients stratified with treatment strategy in the subgroup of mixed LCNEC or early T1–2 tumors (*P* > 0.05).

## Discussion

In the present study, to summarize a comprehensive overview of available data, we conducted an in-depth analysis of 39 cases of LCNEC of the bladder. In our work, we found that the natural history of LCNEC of the bladder differs from that of urothelial carcinoma and the optimal treatment strategy may be different from the urothelial carcinoma of bladder.

Similar to the urothelial carcinoma of bladder, nearly 80% patients were diagnosed after 50 years old with a male-female ratio nearly 4:1. Consistent with previous studies reporting bladder small cell carcinoma (30), the most commonly symptom of LCNEC of the bladder was hematuria (76.9%). Although the impact of race on bladder cancer has not been confirmed so far, LCNEC of the bladder seems to affect Caucasian more frequently. This may be related to the genetic susceptibility, dietary habits and environmental factors in different populations.

Previous studies have shown that tobacco smoking is the most important risk factor for both large cell neuroendocrine carcinoma of the lung and urothelial carcinoma of bladder (31, 32). Consistent with BCa and small cell carcinoma of bladder (33–37), we found that more than 50% of the patients with LCNEC of the bladder were reported as smokers. On the other hand, prior studies have shown that radiation therapy for other pelvic organ tumors may increase the risk of BCa (38, 39). In our study, 3 patients (7.7%) had a history of radiation for extravesical tumor. Among the 3 patients, 2 patients (8, 28) had prostatic cancer treated and other patient (10) had cervical cancer. We speculated that radiation therapy for extravesical tumor may be a risk factor for LCNEC of the bladder. There are many hypotheses about the origin of LCNEC, including pluripotent stem cells, submucosa neuroendocrine cells, or urinary tract epithelial metaplasia (5, 27). Most authors accepted that LCNEC originated from pluripotent stem cells (6, 9, 15, 27). In our study, almost half of the patients (43.6%) of LCNEC of the bladder coexisted with other tumor components, such as UC and SCC, which favors the hypothesis of the origin of multipotent stem cells. Not the same as USCC reported by Zhong et al. (40), there was no difference in OS between the pure LCNEC and mixed LCNEC groups. We think there are two probable reasons why no difference was found between the two groups. The first was the limited sample size and secondly, the biological characteristics of the mixed-type cases may be dominated by the LCNEC component.

Approximately 3/4 of patients with bladder cancer present with non-muscle-invasive bladder cancer (NMIBC), a disease confined to the mucosa (Ta, CIS), or submucosa (T1) (40). However, for LCNEC of the bladder, only 1 (3.3%) patient was classified as NMIBC. Moreover, 20 (66.7%) patients presented advanced tumors (≥pT3) or regional lymph node invasion at the time of diagnosis. Nearly 25% patients were clinically diagnosed with distant metastases before surgery. This finding may be associated with the inconspicuous clinical manifestations of the disease and our data suggested the aggressive natural history of LCNEC of the bladder.

Moreover, the 1-year OS is lower than 55% and the 3-year OS rate is even lower than 25%. Local or distant recurrence were observed in 14 patients out of 27 patients with available data during a relatively short follow-up.

Due to the limited published data for LCNEC of the bladder, there are no standard treatment for the disease. Currently, the treatment strategy for LCNEC of the bladder is similar to that of lung SCNEC (24). Multimodality treatment included surgery, chemotherapy, and radiotherapy were most frequently recommended (41). A recent study analyzed the SEER database and demonstrated that patients with bladder neuroendocrine carcinomas who received cystectomy + chemotherapy + radiotherapy, had the best OS and CSS (5). Consistently, our study demonstrated that multimodality therapy patients had better long-term OS compared to the patients with single nodal treatment. Bhatt et al. (24) reported that platinum-based neoadjuvant chemotherapy can improve the survival rate of neuroendocrine urinary bladder cancer. However, only 3 patients received neoadjuvant chemotherapy in our study.

With regard to the surgical options, our data showed that 71.1% cases were initially treated with diagnostic TURBT and followed by radical cystectomy. However, no statistical difference in OS was shown between the patients in RC group and bladder-sparing group. Moreover, only one patient developed intravesical recurrence among the patients in bladder-sparing group. With this regard, the bladder-sparing approach involving TURBT or partial cystectomy, may be an optional surgical method to improve the quality of life for selected patients with LCNEC of the bladder. Of course, this hypothesis needs to be tested cautiously for limited cases.

Several limitations need to be addressed in our study. First, the results have to be viewed cautiously because of the retrospective nature and small sample size. Second, the study is mainly based on individual case reports or small case series, which may cause heterogeneity of diagnosis and management. Finally, multivariate analysis was not performed because of the limited available data.

## Conclusion

LCNEC of the bladder is an extremely rare tumor with aggressive natural history and poor prognosis. To our knowledge, the present study reported the first case of primary pure LCNEC of the bladder among Chinese patients. Early pathologic stage, and multimodality treatment may be important factors in determining prognosis. However, more in-depth study is needed to better understand the disease.

## Data Availability Statement

The raw data supporting the conclusions of this article will be made available by the authors, without undue reservation.

## Ethics Statement

The studies involving human participants were reviewed and approved by the medical ethical committee of Sun Yat-sen Memorial Hospital, Sun Yat-sen University. The patients/participants provided their written informed consent to participate in this study. Written informed consent was obtained from the individual(s) for the publication of any potentially identifiable images or data included in this article.

## Author Contributions

KX and WZ designed the study and wrote the manuscript. JC, YL, GH, and HL collected and analyzed the clinical data and pathological review. WD, WH, TL, and JC modified and revised the manuscript. JH performed the surgeries and made important revisions to the manuscript. All authors have approved the submission of this manuscript.

## Conflict of Interest

The authors declare that the research was conducted in the absence of any commercial or financial relationships that could be construed as a potential conflict of interest.
