# Supplementary material for: Clinical Characteristics, Treatment Strategy, and Outcomes of Primary Large Cell Neuroendocrine Carcinoma of the Bladder: A Case Report and Systematic Review of the Literature
Source: Front Oncol. 2020 Jul 28;10:1291. doi: 10.3389/fonc.2020.01291 (PMC7399333; doi:10.3389/fonc.2020.01291)
Supplement: Supplementary file 1 [file Data_Sheet_1.PDF]

Supplementary Material

1.Supplementary Figure

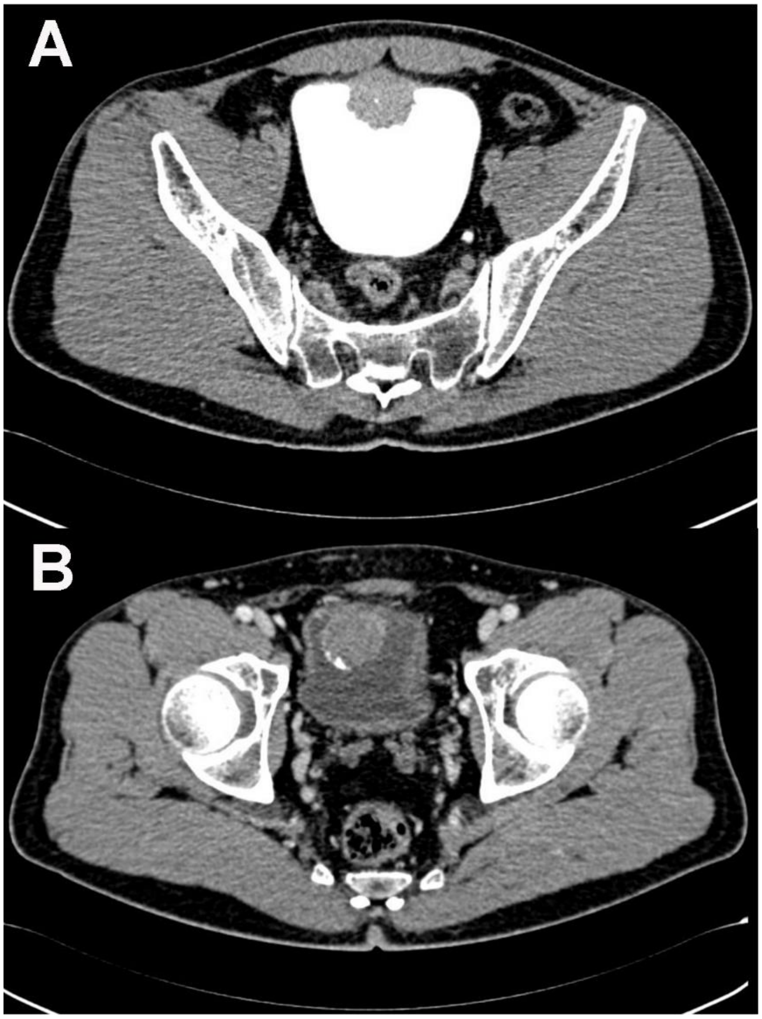

Supplementary Figure 1. Contrast-Enhanced computed tomography (CT) scan of the pelvis revealed a 41.0mm×34.8mm×31.0 mm mass addressing the anterior right wall of bladder. The density of the neoplasm is slightly higher with multiple high-density calcification lesions (A) and moderate enhancement after contrast-enhanced (B).

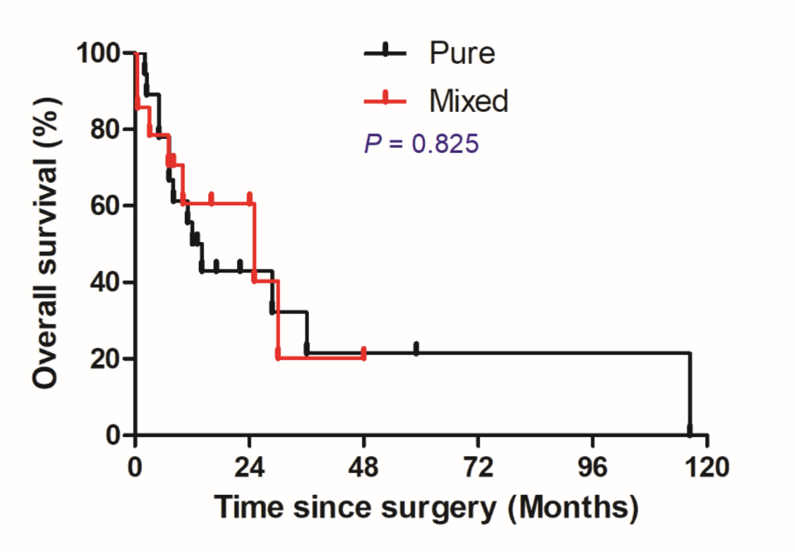

Supplementary Figure 2. Overall Kaplan–Meier survival curves for patients with LCNEC of the bladder stratified according to histology (Pure versus Mixed).

2.Supplementary Table

Supplementary table 1 The baseline of clinicopathologic characteristics and therapy of patients between Bladder-sparing surgery group and RC group.

Characteristics	Bladder-sparing surgery (n = 11)	RC (n = 21)	P-value
**Age (years)**			0.108
≥ 65	8 (72.7%)	9 (42.9%)	
< 65	3 (27.3%)	12 (57.1%)	
**Gender**			0.374
Male	10 (90.9%)	15 (71.4%)	
Female	1 (9.1%)	6 (28.6%)	
**Race**			0.197
Caucasian	7 (63.6%)	18 (85.7%)	
Asian	4 (36.4%)	3 (14.3%)	
**Histology**			0.721
Pure	6 (54.5%)	13 (61.9%)	
Mixed	5 (45.5%)	8 (38.1%)	
**Tumor size (cm)**			0.077
≤ 4	0 (0)	7 (63.6%)	
> 4	4 (100.0%)	4 (36.4%)	
**Pathologic stage**			0.390
T1–2	4 (50.0%)	6 (28.6%)	
T3–4	4 (50.0%)	15 (71.4%)	
**Pathologic node status**			0.638
N0	4 (50.7%)	10 (71.4%)	
N+	3 (42.9%)	4 (28.6%)	
**Distant metastasis at diagnosis**			0.362
M0	5 (62.5%)	13 (81.3%)	
M+	3 (37.5%)	3 (18.8%)	
**Adjuvant chemotherapy**			0.907
Yes	6 (54.5%)	11 (52.4%)	
No	5 (45.5%)	10 (47.6%)	
**Radiotherapy**			0.390
Yes	3 (27.3%)	3 (14.3%)	
No	8 (72.7%)	18 (85.7%)	

Abbreviation: RC, radical cystectomy.

Supplementary table 2 The baseline of clinicopathologic characteristics and therapy between the pure LCNEC and mixed LCNEC groups.

Characteristics	Pure (n = 22)	Mixed (n = 17)	*P*-value
**Age (years)**			0.681
≥ 65	10 (50.0%)	8 (42.9%)	
< 65	10 (50.0%)	6 (57.1%)	
**Gender**			0.709
Male	18 (81.8%)	13 (76.5%)	
**Female**	4 (18.2%)	4 (20.5%)	
**Race**			1
Caucasian	18 (81.8%)	14 (82.4%)	
Asian	4 (18.2%)	3 (17.6%)	
**Tumor Size (cm)**			1
≤ 4	3 (50.0%)	5 (50.0%)	
> 4	3 (50.0%)	5 (50.0%)	
**Pathologic stage**			0.442
T1–2	4 (25.0%)	6 (42.9%)	
T3–4	12 (75.0%)	8 (57.1%)	
**Pathologic node status**			0.397
N0	9 (75.0%)	5 (55.6%)	
N+	3 (25.0%)	4 (44.4%)	
**Pathologic metastasis status**			0.567
M0	2 (40.0%)	4 (66.7%)	
M+	3 (60.0%)	2 (33.3%)	
**Surgery**			0.970
RC	15 (68.2%)	11 (68.8%)	
Bladder-sparing surgery	7 (31.8%)	5 (31.3%)	
**Adjuvant Chemotherapy**			0.332
Yes	15 (68.2%)	9 (52.9%)	
No	7 (31.8%)	8 (47.1%)	
**Radiotherapy**			1
Yes	5 (22.7%)	3 (17.6%)	
No	17 (77.3%)	14 (82.4%)	

Abbreviation: RC, radical cystectomy.
